# Immune biomarkers associated with clinical benefit from atezolizumab (MPDL3280a; anti-PD-L1) in advanced urothelial bladder cancer (UBC)

**DOI:** 10.1186/2051-1426-3-S2-P83

**Published:** 2015-11-04

**Authors:** Thomas Powles, Dorothee Nickles, Eliezer Van Allen, Colombe Chappey, Wei Zou, Marcin Kowanetz, Edward Kadel, Mitchell Denker, Zachary Boyd, Nicholas Vogelzang, Joseph Kim, Joaquim Bellmunt, Yohann Loriot, Charles G Drake, Carol O'Hear, Marcella Fasso, Priti Hegde, Sanjeev Mariathasan

**Affiliations:** 1 Barts Cancer Institute, Queen Mary University of London, London, UK; 2 Genentech, Inc., South San Francisco, CA, USA; 3 Dana-Farber Cancer Institute, Boston, MA, USA; 4 US Oncology Research, Comprehensive Cancer Centers, Las Vegas, NV, USA; 5 Yale Cancer Center, New Haven, CT, USA; 6 Gustav Roussy Institute of Oncology, Paris-Sud University, Paris, France; 7 Department of Oncology, Sidney Kimmel Comprehensive Cancer Center at Johns Hopkins University, Baltimore, MD, USA

## Background

Atezolizumab (anti-PD-L1) has demonstrated robust clinical activity in UBC [1]. Elevated PD-L1 expression on tumor-infiltrating immune cells (IC) is associated with increased clinical efficacy; however, the contribution of other immune biomarkers is unknown. In this study, we explored tumor-based and circulating biomarkers and their correlation with clinical benefit in atezolizumab-treated patients with UBC.

## Methods

Patients from the UBC cohort (n = 92) of the Phase Ia atezolizumab trial PCD4989g (NCT01375842) served as source population for tumor specimens, plasma and PBMC. Baseline tumor PD-L1 expression was assessed by immunohistochemistry using the SP142 antibody assay optimized to detect PD-L1 on both tumor cells (TC) and IC. RNA gene expression on tumor and PBMC samples was interrogated with a NanoString panel of 800 immune and cancer genes. Sequential blood draws assessed dynamic changes in circulating immune biomarkers in plasma (RBM, Multi-Analyte Platform). Correlation between biomarker expression and 6-month progression-free survival (PFS; as a measure of clinical benefit) was assessed.

## Results

Baseline gene expression in tumors revealed an effector T cell signature (including *CD8A, GZMA, IFNG*) and NK gene signature (*NKG2* family members) associated with clinical benefit. In contrast, disease progression was associated with either a concomitant presence of the immune signature and an opposing stromal signature (*PDPN, COL5A1*, etc) or the absence of both signatures. Expression of T cell effector and immune checkpoint genes (*CTLA4, PD-1, TIGIT, LAG3*) correlated with PD-L1 expression on IC but not TC. Increased expression of myeloid-derived cytokines (IL-6 and IL-8) in the plasma was associated with lack of clinical benefit. Moreover, on-treatment sampling revealed an increased plasma HCG, CA15-3 and TIMP-1 to be correlated with disease progression. Immune biomarkers associated with PBMC, as well as tumor biomarkers associated with various tumor subtypes, will also be discussed.

## Conclusions

Our findings indicate that clinical benefit (as defined by 6-month PFS) from atezolizumab is influenced by a pre-existing CD8+ effector T cell and NK cytolytic gene signature in the tumor, which correlated with IC PD-L1 expression. Increased stromal and myeloid-derived cytokine expression in tumor and plasma, respectively, were associated with lack of clinical benefit, underscoring the complex interplay among immunological components in UBC. These components may be conceivable targets to overcome potential resistance and promote response to atezolizumab.

ClinicalTrials.gov Identifier: NCT01375842
